# Comprehensive Pan-Cancer Genomic Analysis Reveals PHF19 as a Carcinogenic Indicator Related to Immune Infiltration and Prognosis of Hepatocellular Carcinoma

**DOI:** 10.3389/fimmu.2021.781087

**Published:** 2022-01-05

**Authors:** Zheng-yi Zhu, Ning Tang, Ming-fu Wang, Jing-chao Zhou, Jing-lin Wang, Hao-zhen Ren, Xiao-lei Shi

**Affiliations:** ^1^ Department of Hepatobiliary Surgery, Affiliated Drum Tower Hospital of Nanjing University Medical School, Nanjing, China; ^2^ Nanjing Drum Tower Hospital Clinical College of Nanjing Medical University, Nanjing, China; ^3^ Nanjing Drum Tower Hospital Clinical College of Traditional Chinese and Western Medicine, Nanjing University of Chinese Medicine, Nanjing, China

**Keywords:** PHD finger protein 19 (PHF19), pan-cancer, immune infiltration, prognosis, The Cancer Genome Atlas (TCGA), hepatocellular carcinoma (HCC), predictive model, tumor microenvironment (TME)

## Abstract

**Background:**

As a crucial constituent part of Polycomb repressive complex 2, PHD finger protein 19 (PHF19) plays a pivotal role in epigenetic regulation, and acts as a critical regulator of multiple pathophysiological processes. However, the exact roles of PHF19 in cancers remain enigmatic. The present research was primarily designed to provide the prognostic landscape visualizations of PHF19 in cancers, and study the correlations between PHF19 expression and immune infiltration characteristics in tumor microenvironment.

**Methods:**

Raw data in regard to PHF19 expression were extracted from TCGA and GEO data portals. We examined the expression patterns, prognostic values, mutation landscapes, and protein-protein interaction network of PHF19 in pan-cancer utilizing multiple databases, and investigated the relationship of PHF19 expression with immune infiltrates across TCGA-sequenced cancers. The R language was used to conduct KEGG and GO enrichment analyses. Besides, we built a risk-score model of hepatocellular carcinoma (HCC) and validated its prognostic classification efficiency.

**Results:**

On balance, PHF19 expression was significantly higher in cancers in comparison with that in noncancerous samples. Increased expression of PHF19 was detrimental to the clinical prognoses of cancer patients, especially HCC. There were significant correlations between PHF19 expression and TMB or MSI in several cancers. High PHF19 levels were critically associated with the infiltration of myeloid-derived suppressor cells (MDSCs) and Th2 subsets of CD4+ T cells in most cancers. Enrichment analyses revealed that PHF19 participated in regulating carcinogenic processes including cell cycle and DNA replication, and was correlated with the progression of HCC. Intriguingly, GSEA suggested that PHF19 was correlated with the cellular components including immunoglobulin complex and T cell receptor complex in HCC. Based on PHF19-associated functional gene sets, an eleven-gene prognostic signature was constructed to predict HCC prognosis. Finally, we validated pan-cancer PHF19 expression, and its impacts on immune infiltrates in HCC.

**Conclusion:**

The epigenetic related regulator PHF19 participates in the carcinogenic progression of multiple cancers, and may contribute to the immune infiltration in tumor microenvironment. Our study suggests that PHF19 can serve as a carcinogenic indicator related to prognosis in pan-cancer, especially HCC, and shed new light on therapeutics of cancers for clinicians.

## Introduction

Cancer is a major concern regarding public health and the primary cause of death worldwide, and the incidence and mortality are rapidly increasing globally (1). Although cancer treatment has improved substantially over the last decades and currently allows cures for many previously fatal cases, large quantities of patients still experienced therapeutic failure and succumbed to cancer (2). Accordingly, there is a dire need to clarify the molecular mechanisms elucidating patterns of cancer pathogenesis and to identify reliable biomarkers for the early detection, diagnosis and treatment of cancers (3). Since the first human genome sequencing in 2001, comprehensive genomic characterization of tumors has become a major goal in the field of cancer research, and recent advances in sequencing technologies and computational analytical methods have revolutionized cancer research studies (4). Large-scale genomics projects like The Cancer Genome Atlas (TCGA) database, and the public repository named the NCBI Gene Expression Omnibus (GEO), provide matched molecular and clinical data of various cancers, which helps systematically analyze the survival impact of single gene expression. Currently, the application of cancer biomarkers has aroused great interest among scientists, which encourages researchers to explore novel prognostic biomarkers and therapeutic targets.

Polycomb group (PcG) proteins, as a class of widely-studied epigenetic modifiers, form large multiprotein complexes that serve as chromatin-modifying or -remodeling enzymes and participate in maintaining cell identity and cell differentiation, by keeping the transcriptional repression of functional genes which regulate developmental processes or cell-cycle progression (5, 6). Dysregulation of PcG proteins was reported to play pivotal roles in the anomalous activation of cellular differentiation, carcinogenesis, cancer development and progression (7). PcG proteins generally assemble in two functionally distinct Polycomb repressive complexes (PRCs) referred to as PRC1 (responsible for H2AK119 monoubiquitylation) and PRC2 (catalyzing H3K27 methylation) (8). The PRC2 core formed by enhancer of zeste homolog 1/2 (EZH1/2), suppressor of zeste 12 (SUZ12), the embryonic ectoderm development (EED) and retinoblastoma-binding protein 4/7 (RBBP4/7), can interact with several substoichiometrical accessory proteins that modulate its function, including Polycomb-like (PCL) proteins (9, 10). The *Pcl* gene was initially identified in *Drosophila melanogaster* (11), and three mammalian homologs of *Drosophila Pcl* have been characterized to date, termed PCL1 [also named PHD finger protein 1 (PHF1)], PCL2 [also named Metal response element binding transcription factor 2 (MTF2)], and PCL3 [also named PHD finger protein 19 (PHF19)], respectively (12). These PCL proteins are PRC2-relevant factors that form sub-complexes with PRC2 core components, and regulate the enzyme activity of PRC2 or its recruitment to the target loci (13).

PHD finger protein 19 (PHF19), namely PCL3, is an critical component of PRC2 that acts as a transcriptional repressor of several developmentally regulated genes and functions as a pivotal regulator of various biological processes (14). PHF19 protein contains a single Tudor domain followed by two plant homeodomain (PHD) fingers and an extended homologous (EH) domain, and binds trimethylated histone H3 Lys36 (H3K36me3) through its Tudor domain (15, 16). Direct recognition of H3K36me3 by PHF19 is a requisite for the complete enzyme activity of PRC2 complex and serves to recruit PRC2 and H3K36me3 demethylases NO66 or KDM2b to specific genomic loci to facilitate the removal of H3K36me3 active mark and deposition of histone H3 Lys27 trimethylation (H3K27me3) (16, 17). Previous researches have elucidated that PHF19 is overexpressed in multiple cancerous tissues compared with the normal tissue counterparts. For instance, PHF19 expression is present in all subgroups of multiple myeloma (MM) and is preferentially upregulated in high-risk MM (18). Aberrant overexpression of PHF19 has also implicated in gastric cancer, associated with cancer cell differentiation and poor prognosis for patients (19). Significantly elevated in the advanced stages of Glioblastoma (GBM), PHF19 was reported to block the degradation of β-catenin *via* transcriptional repression of SIAH1 and promote the progression of GBM (20). Tissue microarray analysis of surgically resected paired colorectal cancer (CRC) samples showed that PHF19 protein was overexpressed in CRC tissues compared with paired adjacent normal tissues (21). Nevertheless, despite the efforts to understand the roles of PHF19 in multiple cancers, a comprehensive analysis that determines the genetic targets and mis-regulated pathways controlled by PHF19 has not been reported so far, and the molecular contributions of PHF19 remain elusive.

In the current research, we conducted a integrative pan-cancer analysis of tumor samples from public databases. We investigated the expression patterns of PHF19 in normal tissues, various cell lines and cancers, and estimated the prognostic values of PHF19 in pan-cancer based on multiple databases. Besides, we explored the links between PHF19 expression and tumor mutation burden (TMB), microsatellite instability (MSI), immune checkpoints and immune infiltration, and identified the specific genes and signaling pathways involved in the regulation of cancer development by PHF19. Finally, due to the fact that functional enrichment analysis of PHF19 was obviously correlated with hepatocellular carcinoma (HCC), we constructed a PHF19-related prognostic risk-score model for HCC patients and performed a validation of this model in an external dataset. These findings may have important implications in guiding basic research as well as clinical practice.

## Materials And Methods

### Data Acquisition and Processing

As a landmark cancer genomics project, TCGA molecularly characterized more than 20,000 primary cancer and corresponding normal tissues across 33 cancer types (22). In our analysis, TCGA transcriptome RNA-seq data and clinical information were downloaded using the UCSC Xena platform (23). Transcripts per million (TPM) and fragments per kilobase million (FPKM) were used for quantification and comparison. Besides, two liver hepatocellular carcinoma (LIHC) cohorts and matched clinical data used in our study were respectively obtained from the Genomic Data Commons (GDC) Data Portal (https://portal.gdc.cancer.gov/) and the GSE14520 dataset from the GEO database (24).

### Patients and Clinical Specimens

All the biospecimens are provided by Nanjing multicenter biobank, biobank of Nanjing Drum Tower Hospital, the Affiliated Hospital of Nanjing University Medical School. Written informed consents were obtained from all subjects, and normalized ethnic audit has been proceeded.

### Reagents

Antibody recognizing PHF19 (Proteintech, 11895-1-AP) was purchased from Proteintech. For flow cytometry analysis, antibodies against CD14 (clone M5E2, 301808), CD11b (clone M1/70, 101205), CD33(clone WM53, 303404), CD4 (clone OKT4, 317416), IL-4 (clone MP4-25D2, 500806) were purchased from BioLegend. For RT-qPCR, the primers were as follows: PHF19 forward primer 5’-ACTCGGGACTCCTATGGTGC-3’, reverse primer 5’-CCTCCGTCAGTTTGGACATCA-3’; and GAPDH forward primer 5’-GGAGCGAGATCCCTCCAAAAT-3’, reverse primer 5’-GGCTGTTGTCATACTTCTCATGG-3’.

### Analysis of PHF19 mRNA Expression Profiles

The mRNA expression profiles of PHF19 in major tissues and organs of human body were explored in the Human Protein Atlas (HPA), as well as the single cell transcriptomics analysis (25). Transcript levels of PHF19 in different cancers were analyzed using the ONCOMINE database (26), under the settings of P-value = 0.001 and fold change (FC) = 1.5, and in the “Gene_DE” module of TIMER2.0 database (27). Differential mRNA expression analysis of normal and tumor samples, and pathological stage analysis of PHF19, were performed in the “Single Gene Analysis” module of GEPIA (28). “Expression on Box Plots” module was used to depict box plots of expression differences between tumors and matched normal samples of the GTEx database, with the thresholds set as a P-value cutoff of 0.01 and log_2_FC cutoff of 1, and “Match TCGA normal and GTEx data” was set. The log_2_(TPM + 1) data was applied for log-scale.

### Survival Analysis

Cox regression analysis for TCGA datasets was performed using RStudio software (version 1.2.5042) with the “survival” and “forestplot” package to investigate the correlation between PHF19 expression and cancer prognosis, including overall survival (OS) and disease-specific survival (DSS). We calculated the log-rank P-value and hazard ratio (HR) with 95% confidence intervals (95% CI) *via* the “survival” package and utilized the “forestplot” package to visualize the survival analysis. The Kaplan-Meier plotter, which is a web database aiming to evaluate the effect of 54,000 genes on survival in 21 tumor types (29), was used to determine PHF19 expression-associated OS outcomes of patients. Additionally, the GEPIA2 database was also utilized to determine the correlation between PHF19 mRNA expression and OS and disease-free survival (DFS) of cancers (30).

### Genomic Alterations and Mutation Profiles

Based on the cBioPortal tool (http://www.cbioportal.org/), PHF19 mutation frequency and general mutation count in cancer patients were calculated to analyze the genomic alterations of PHF19 in various TCGA cancer types (31). The genome alterations of PHF19 included copy number amplification, deep or shallow deletion, missense mutation with uncertain significance and mRNA upregulation. Tumor mutation burden (TMB) is calculated as total somatic nonsynonymous mutation counts in coding regions and emerging as a biomarker for predicting immunotherapy effect. Microsatellite instability (MSI) refers to the nucleotide insertions or deletions in the microsatellite loci. The TMB and MSI scores were obtained from TCGA database and analyses regarding association between PHF19 expression and TMB or MSI were conducted by R language.

### Immune Infiltration Analysis

The ESTIMATE algorithm (32), which is a method that infers the fraction of immune and stromal cells in tumor samples *via* analysis of gene expression signatures, was applied to evaluate the immune cell infiltration levels (ImmuneScore) and the abundance of stromal components (StromalScore) for each TCGA sample in the RStudio software with the “estimate” package. The relationships of PHF19 expression with immune or stromal scores in several cancers were visualized as scatter plots. Higher ImmuneScore or StromalScore indicated larger proportion of immune or stromal components in tumor microenvironment (TME).

“Gene_Corr” module of TIMER2.0 database was utilized to explore the correlations between PHF19 expression and immune checkpoint-associated genes, including BTLA, CD27, CD274, CD276, CD28, CD40, CD70, CD80, CD86, CTLA4, HAVCR2, HHLA2, ICOS, ICOSLG, IDO1, IDO2, LAG3, PDCD1, TIGIT, TNFRSF9, and TNFSF9 across human cancers from the TCGA cohorts (33). The generated heatmap suggested statistical significance and provided the purity-adjusted partial Spearman’s rho value, which avoided the effect of outliers. Besides, “Immune-Gene” tool of TIMER2.0 database was applied to explore the association between PHF19 level and immune cell infiltration in all TCGA cancers. Immune cells including myeloid-derived suppressor cells (MDSCs), Th1 and Th2 subsets of CD4+ T cells were selected. The TIDE and XCELL algorithms were applied to estimate the immune infiltration and the results were depicted as a heatmap and scatter plots.

### Enrichment Analysis

The protein-protein interaction (PPI) network was established applying the Search Tool for the Retrieval of Interacting Genes (STRING) with the following input parameters: “evidence”, “experiments”, and 0.200 confidence level (34). The protein interaction file from STRING database was imported into the Cytoscape software (version 3.8.2) for PPI network construction, visualization and analysis (35). Besides, we adjusted the parameter “minimum required interaction score” to conformity = 0.150 and set the parameter “max number of interactors to show” as “no more than 50 interactors”, in order to get access to experimentally determined PHF19-binding proteins.

“Similar Genes Detection” function of GEPIA2 database was utilized to acquire the first 100 PHF19-correlated genes based on the TCGA and GTEx datasets. “Correlation Analysis” module of GEPIA2 was applied to compute pair-wise gene expression correlations between PHF19 and selected genes, using the Pearson correlation method. We also used the “Gene_Corr” function of TIMER2.0 database to acquire the heatmap data of corresponding genes, containing the partial correlation coefficient (cor) and P-value calculated by the purity-adjusted Spearman’s rank correlation test. Meanwhile, the Venn diagram was generated by a Venn diagram tool (http://bioinformatics.psb.ugent.be/webtools/Venn/) to perform the intersection analysis of the PHF19-binding and associated genes. These two sets of genes were combined to perform Kyoto Encyclopedia of Genes and Genomes (KEGG) pathway enrichment analysis by Metascape portal, which is designed to offer a comprehensive gene list annotation and analysis resource for experimental biologists (36). The resulting enriched pathways were visualized using the “ggplot2” R package. Besides, we conducted the Gene Ontology (GO) analysis to access the molecular functions (MF) *via* the “clusterProfiler” R package and the result was visualized using the cnetplot function.

CancerSEA is a dedicated database that portrays single-cell functional status maps that involve fourteen functional states of more than 40,000 single cells across 25 cancer types, aiming at comprehensively decoding distinct functional states of cancer cells at single-cell resolution (37). In the present research, the CancerSEA database was applied for the functional analysis of PHF19. Further gene set enrichment analysis (GSEA) was performed to identify the significant pathways between low expression and high expression group of PHF19, and the top four terms of GO analysis and transcription factor targets were exhibited, using the “clusterProfiler” R package.

### Construction and Evaluation of Prognostic Risk Model

Forty genes were extracted from the PHF19 functionally associated gene set obtained by GO-MF analysis as described above, and the “limma” R package was used to determine differentially expressed genes (DEGs) between TCGA HCC samples and normal controls. We conducted univariate and multivariate Cox regression analyses by the “survival” package, and performed LASSO regression using the “glmnet” package to acquire the most useful predictive genes. The risk assessment model was constructed based on the corresponding coefficients and then applied to patients to generate the risk score of each patient. Patients in TCGA LIHC cohort and GSE14520 cohort were respectively separated into high- and low-risk groups in accordance with the median value of risk scores. We evaluated the predictive capability of the risk model by survival analysis and Receiver Operating Characteristic (ROC) curves. Univariate and multivariate Cox regression analyses were then conducted to confirm the prognostic efficiency of the risk-score model, as well as other clinicopathological features. Nomograms were formulated by using the “rms” R package in RStudio.

### Statistical Analysis

For experimental studies, at least three biological replicates were repeated. Data were shown as average values ± SEM. The P value was calculated using GraphPad Software.

## Results

### PHF19 Expression Profiles in Human Normal Tissues and Cancers

**Figure 1:**
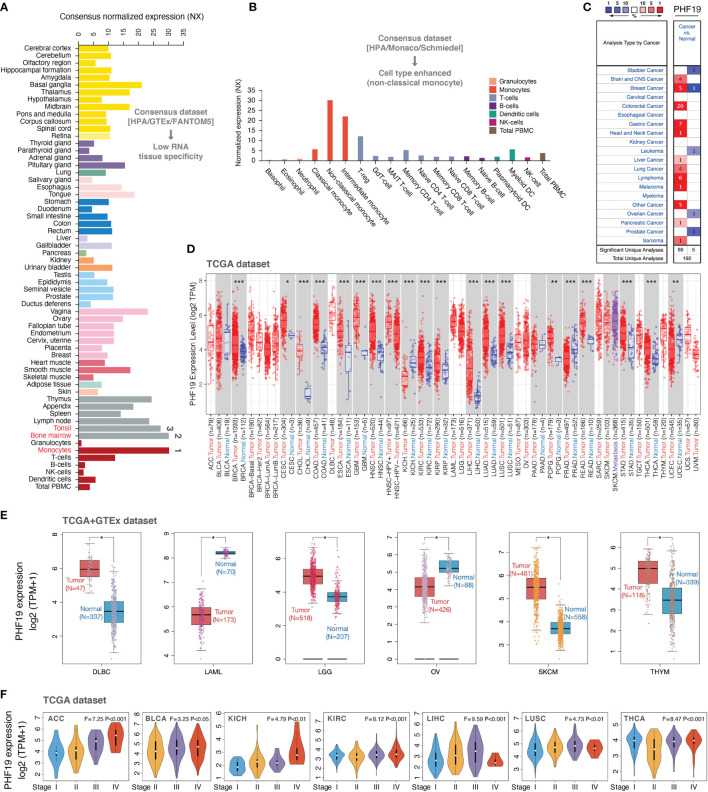
PHF19 expression profiles in normal tissues and cancers. **(A)** PHF19 expression levels in normal tissues and cell types. **(B)** PHF19 expression levels in blood cell types. **(C)** Transcription levels of PHF19 in datasets of multiple cancers compared with noncancerous tissues. The figure was generated from the ONCOMINE database. **(D)** Expression levels of PHF19 in TCGA cancers were analyzed by TIMER2.0 database (*P < 0.05; **P < 0.01; ***P < 0.001). **(E)** Differences of PHF19 expression between cancers from the TCGA database and normal samples from the GTEx database (*P < 0.05). **(F)** PHF19 expression levels were assessed by the main pathological stages of ACC, BLCA, KICH, KIRC, LIHC, LUSC, and THCA. The log_2_(TPM + 1) for log-scale was used.

To determine the expression profiles of PHF19 in human normal tissues, we investigated the mRNA expression patterns of PHF19 in various non-tumor tissues and single cell types based on publicly available genome-wide expression data. As shown in **Figure 1A**, among all detected tissues and cell types, the highest PHF19 expression was observed in the monocytes, followed by the bone marrow and tonsil, based on the Consensus dataset created by integrating the data from three transcriptomics datasets (HPA, GTEx and FANTOM5). Low RNA tissue specificity was indicated since PHF19 was expressed in all tissues tested, with the consensus normalized expression (NX) in the vast majority of tissues > 1. With regard to RNA blood cell type specificity, interestingly, the PHF19 mRNA expression was obviously enriched in non-classical monocytes, when analyzing in the HPA/Monaco/Schmiedel datasets (**Figure 1B**). Non-classical monocytes express CD14^low^CD16^+^ antigen and constitute about 10%-15% of blood monocytes (38). Our result indicated that PHF19 might be implicated in specific functions of these monocytes.

We next retrieved PHF19 mRNA expression levels over a cancer-wide range *via* the ONCOMINE database. The results determined that compared with that in the corresponding normal groups, PHF19 expression was higher in cancer tissues, such as brain and CNS cancer, breast cancer, colorectal cancer, gastric cancer, head and neck cancer, liver cancer, lung cancer, lymphoma, melanoma, pancreatic cancer, and sarcoma (**Figure 1C**). Yet in certain studies, PHF19 expression was lower in bladder cancer, breast cancer, leukemia, ovarian cancer, and prostate cancer. To further evaluate the expression status of PHF19 spanning various cancer types, we analyzed the TCGA RNA sequencing data by applying the TIMER2.0 approach. As presented in **Figure 1D**, PHF19 expression was significantly elevated in multiple cancer types, including BRCA (breast invasive carcinoma), CESC (cervical and endocervical cancer), CHOL (cholangiocarcinoma), COAD (colon adenocarcinoma), ESCA (esophageal carcinoma), GBM (glioblastoma multiforme), HNSC (head and neck cancer), KIRC (kidney renal clear cell carcinoma), KIRP (kidney renal papillary cell carcinoma), LIHC (liver hepatocellular carcinoma), LUAD (lung adenocarcinoma), LUSC (lung squamous cell carcinoma), PCPG (pheochromocytoma and paraganglioma), READ (rectum adenocarcinoma), STAD (stomach adenocarcinoma), and THCA (thyroid carcinoma), compared with their corresponding adjacent non-cancerous tissues. Meanwhile, PHF19 expression was markedly decreased in KICH (kidney chromophobe), PRAD (prostate adenocarcinoma), and UCEC (uterine corpus) than in their respective normal samples. By integrating data from the GTEx database as normal controls, we further performed differential-expression analysis of PHF19 between tumor and normal samples of DLBC (diffuse large B-cell lymphoma), LAML (acute myeloid leukemia), LGG (lower grade glioma), OV (ovarian serous), SKCM (skin cutaneous melanoma), and THYM (thymoma) (**Figure 1E**). Besides, we further evaluated the correlation of PHF19 expression with cancer pathological stages, including ACC (adrenocortical carcinoma), BLCA (bladder urothelial carcinoma), KICH, KIRC, LIHC, LUSC, and THCA (**Figure 1F**). The results determined a positive relationship between PHF19 level and advanced tumor stages.

### Multifaceted Prognostic Analysis of PHF19 in Cancers

**Figure 2:**
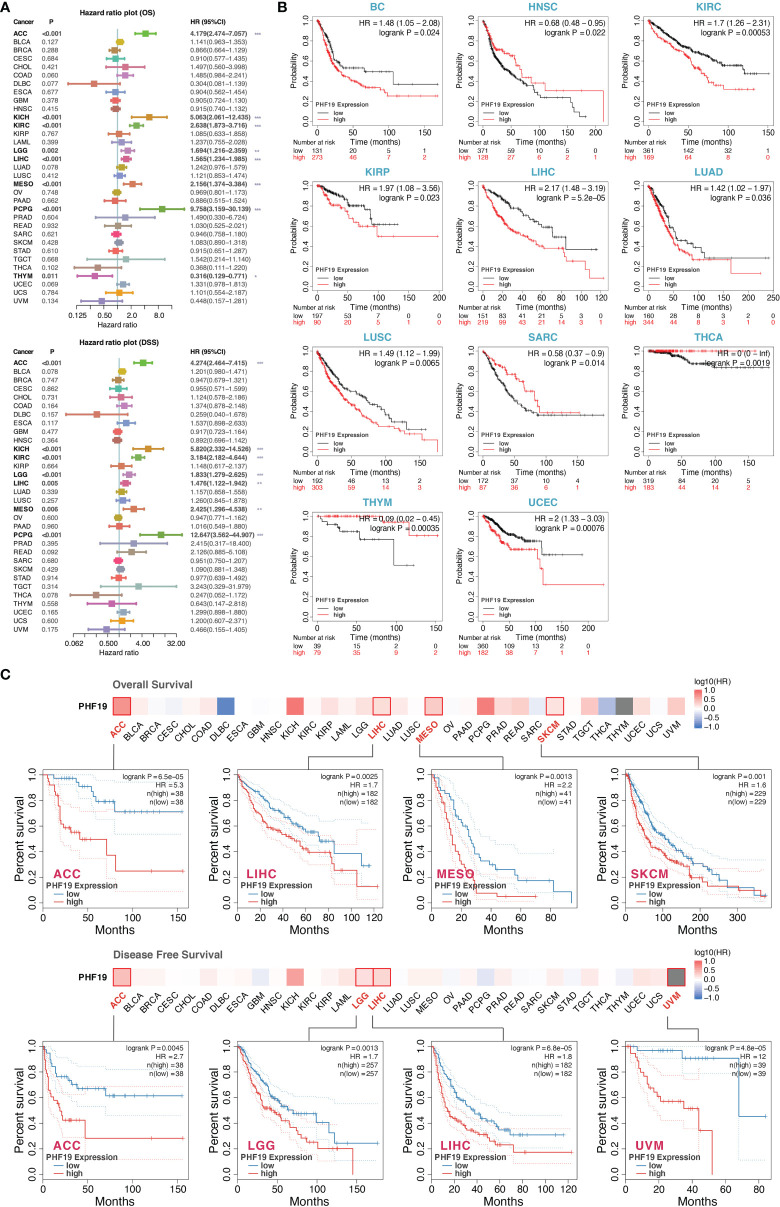
Multifaceted prognostic analysis of PHF19 in cancers. **(A)** Correlations of PHF19 expression with OS and DSS of patients using the Cox regression survival analysis (*P < 0.05; **P < 0.01; ***P < 0.001). **(B)** KM survival curves showed that PHF19 expression was highly associated with clinical outcomes in different cancers. **(C)** The survival maps and survival curves were depicted to perform OS and DFS analyses in cancers.

To investigate the clinical significance of PHF19 in cancer patients, we downloaded the TCGA mRNA sequencing and clinical information of 33 cancer types from the UCSC Xena platform and calculated the correlations of PHF19 expression with overall survival (OS) and disease-specific survival (DSS) of patients using the univariate Cox survival analysis. As shown in **Figure 2A**, the forest plots suggested that elevated PHF19 expression was significantly associated with worse OS in ACC (HR = 4.18, P < 0.001), KICH (HR = 5.06, P < 0.001), KIRC (HR = 2.64, P < 0.001), LGG (HR = 1.69, P = 0.002), LIHC (HR = 1.57, P < 0.001), MESO (mesothelioma) (HR = 2.16, P < 0.001), and PCPG (HR = 9.76, P < 0.001) patients, and also clearly correlated with worse DSS in ACC (HR = 4.27, P < 0.001), KICH (HR = 5.82, P < 0.001), KIRC (HR = 3.18, P < 0.001), LGG (HR = 1.83, P < 0.001), LIHC (HR = 1.48, P = 0.005), MESO (HR = 2.43, P = 0.006), and PCPG (HR = 12.65, P < 0.001) patients. These data showed that high expression of PHF19 was strongly associated with poor patient outcomes in multiple cancer types, which suggested that PHF19 may serve as a potential prognostic biomarker in pan-cancer. Of note, and in contrast, increased PHF19 expression was implicated in prolonged OS in THYM (HR = 0.32, P = 0.011).

Kaplan-Meier (KM) survival curves comparing PHF19 high and low expressing patients were also constructed to further evaluate the prognostic potential of PHF19 *via* the Kaplan-Meier plotter database. The results revealed that high PHF19 expression predicted worse OS in BC (bladder carcinoma), KIRC, KIRP, LIHC, LUAD, LUSC, and UCEC, nevertheless, patients with higher PHF19 expression showed remarkably improved OS in HNSC, SARC, THCA, and THYM (all log-rank P values < 0.05) (**Figure 2B**). We next compared the survival contribution of PHF19 in multiple cancer types, estimated using Mantel-Cox test through the GEPIA2 database, and the survival maps accompanied with OS curves and disease-free survival (DFS) curves are presented in **Figure 2C**. High transcriptional levels of PHF19 were linked to unfavorable prognosis in OS of ACC, LIHC, MESO, and SKCM, and DFS analysis data showed that elevated PHF19 level was related to unfavorable prognosis for ACC, LGG, LIHC, and UVM (uveal melanoma) (all log-rank P values < 0.05). Overall, the above data indicated that PHF19 expression was significantly correlated with patient prognosis in various cancers, especially in LIHC, and the relevance of PHF19 to clinical outcomes may shed new light on the underlying pathogenesis of different tumors.

### Mutation Landscape of PHF19 in Cancers

**Figure 3:**
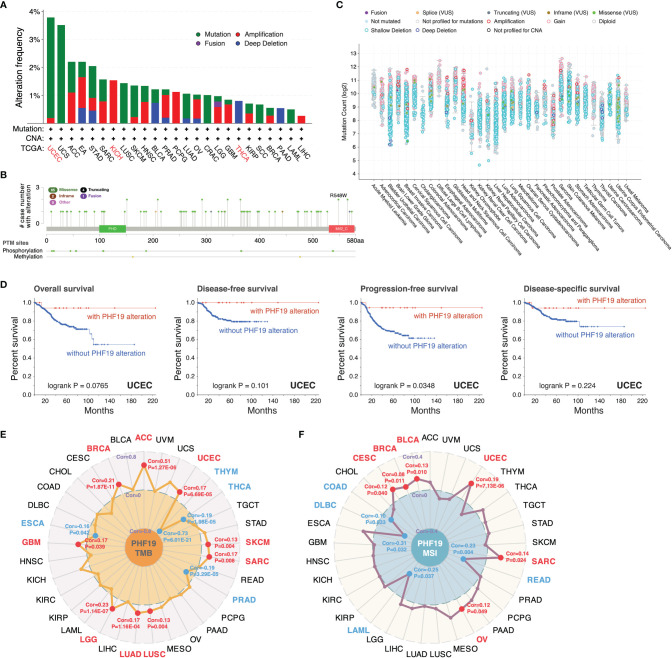
Mutation landscape of PHF19 in cancers. PHF19 alteration frequencies in various cancers **(A)** and mutation sites **(B)** were visualized. **(C)** The general mutation counts of PHF19 in TCGA samples. **(D)** Correlations between PHF19 alteration status and OS, DFS, PFS, and DSS of UCEC. Radar maps of correlations between PHF19 expression and TMB **(E)** or MSI **(F)** were plotted.

We inspected the genomic alterations and mutation profiles of PHF19 in the TCGA cancer cohorts by employing the cBioPortal database. As presented in **Figure 3A**, the highest alteration frequency of PHF19 appeared in UCEC patients with “mutation” as the predominant type, while the “amplification” type of copy number alteration (CNA) and copy number “deep deletion” were respectively the primary type in KICH and THCA. Besides, we detected altogether 72 mutation sites including 65 missense, 4 truncating, 2 inframe, and 1 fusion mutation between amino acids 0 and 580, and the types, sites and case number of PHF19 genomic alterations were shown in **Figure 3B**. We also analyzed the general mutation count of PHF19 in 10953 patients/10967 samples from TCGA datasets (**Figure 3C**). In addition, we investigated the association between PHF19 alteration and the clinical outcomes of UCEC cases, and found that UCEC patients with altered PHF19 showed improved prognosis in terms of progression-free survival (PFS) (log-rank P = 0.035), but not OS (log-rank P = 0.077), DFS (log-rank P = 0.101), and DSS (log-rank P = 0.224), compared with those without PHF19 alteration (**Figure 3D**). Meanwhile, since TMB and MSI are regarded as critical factors impacting on oncogenesis and progression of tumors, and affecting response to immunotherapy in cancers, we next performed association analyses between PHF19 expression and TMB/MSI spanning all TCGA tumor types. As shown in **Figure 3E**, PHF19 expression was positively correlated with TMB in ACC, BRCA, GBM, LGG, LUAD, LUSC, SARC, SKCM, and UCEC, while negatively correlated with TMB in ESCA, PRAD, THCA, and THYM cohorts (all P-values < 0.05). PHF19 expression was also positively correlated with MSI of BLCA, BRCA, CESC, OV, SARC, and UCEC, but negatively correlated with that of COAD, DLBC, LAML, and READ (all P-values < 0.05) (**Figure 3F**). These results may deserve further in-depth investigations.

### PHF19 Expression Correlates With Tumor Immune Infiltration

**Figure 4:**
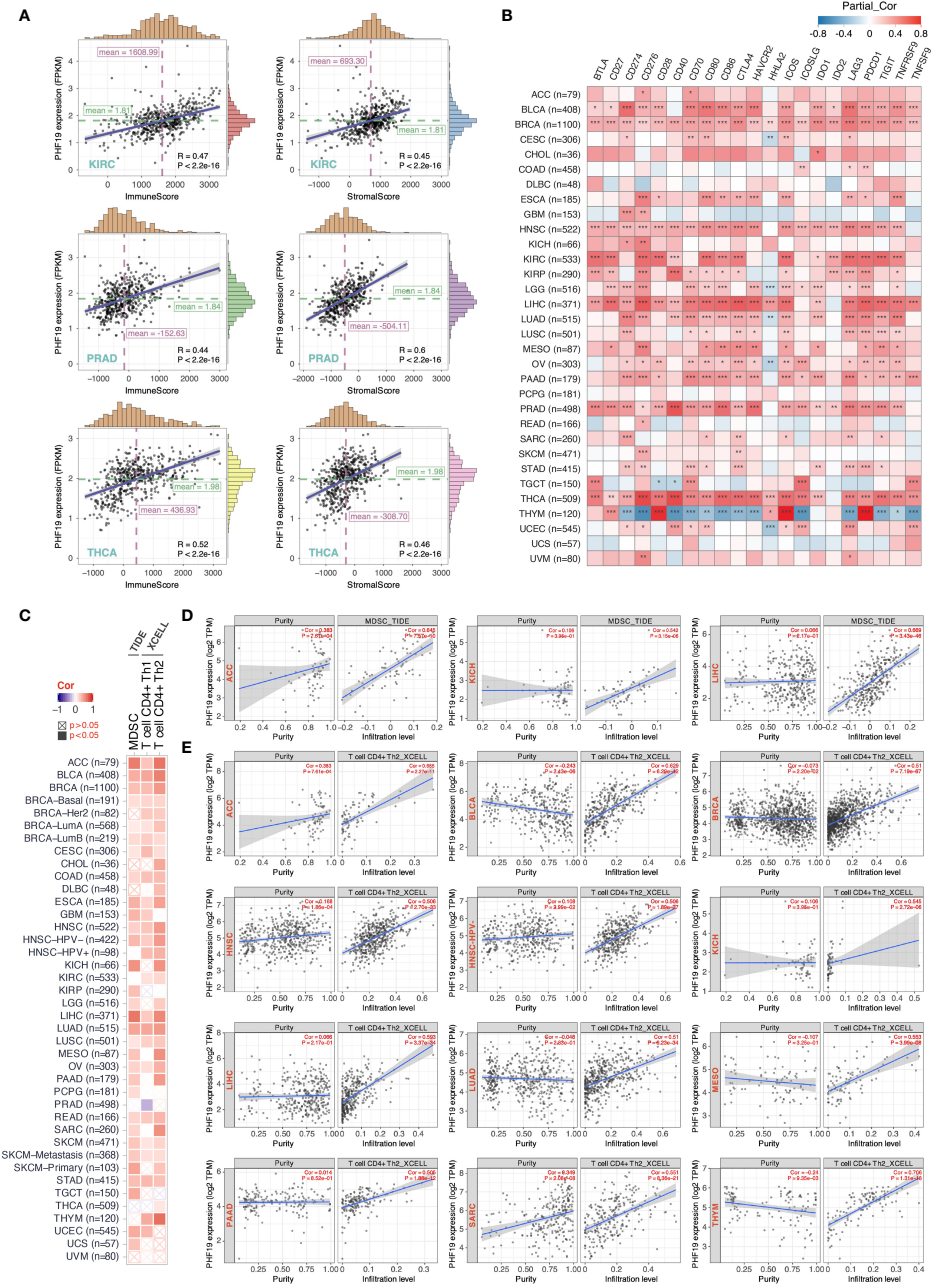
PHF19 expression correlates with the immune infiltrates of tumors. **(A)** Top three scatter plots of correlation between PHF19 expression and immune and stromal scores in multiple cancers. **(B)** Correlations between PHF19 expression level and immune checkpoint-associated genes. **(C)** Correlations between PHF19 expression level and the infiltration level of MDSCs, Th1 and Th2 subsets of CD4+ T cells across TCGA cancers. Scatter plots of MDSC **(D)** and CD4+ Th2 cell **(E)** infiltration level related to PHF19 expression were presented.

Tumor-infiltrating immune cells, as principal compositions of the TME, are frequently involved in tumor behaviors including cancer initiation, progression or metastasis, and are deemed as independent predictors of sentinel lymph node status and cancer prognosis (39). Given that PHF19 expression correlates with TMB and MSI which affect response to cancer immunotherapy, we next explored the correlations between PHF19 expression level and the abundance of immune cell infiltrates. By adopting the ESTIMATE method, we first computed the immune and stromal scores of cancer tissues. As **Figure 4A** indicated, PHF19 was correlated with the immune and stromal scores in KIRC, PRAD and THCA (all data P-values < 0.001). Since immune checkpoint-associated genes participate in the immunosuppressive mechanism that allows tumor cells to escape anti-tumor immunity (40), we next investigated the correlations between PHF19 expression and immune checkpoint-related genes, including BTLA, CD27, CD274, CD276, CD28, CD40, CD70, CD80, CD86, CTLA4, HAVCR2, HHLA2, ICOS, ICOSLG, IDO1, IDO2, LAG3, PDCD1, TIGIT, TNFRSF9, and TNFSF9 across human cancers from the TCGA cohorts, as shown in **Figure 4B**. Our results suggested that PHF19 expression was closely associated with almost all immune checkpoint-associated genes in BLCA, BRCA, HNSC, LIHC, PRAD and THCA, implying that PHF19 might conduce to immune escape in these tumors. Further, we calculated the correlation coefficients of PHF19 expression and immune infiltration levels by employing the TIDE and XCELL algorithms, and depicted the landscape of PHF19 correlating with immune cell infiltrates in various TCGA cohorts. The heatmap exhibited that PHF19 expression was positively and statistically significantly correlated with the immune infiltration of myeloid-derived suppressor cells (MDSCs) and Th2 subset of CD4+ T cells in the majority of cancers (**Figure 4C**). Intriguingly, PHF19 expression was also positively relevant to the infiltration abundance of CD4+ Th1 cells in 18 cancer types, with all correlation coefficients < 0.45, and negatively relevant to that in PRAD, with the correlation coefficient = -0.29. Representative scatter plots of MDSC infiltration level related to PHF19 expression were presented in **Figure 4D**, using the TIDE algorithm (with the correlation coefficient > 0.5). The results indicated that PHF19 expression was obviously positively correlated with the infiltration abundance of MDSCs in ACC (Cor = 0.645, P = 7.57e-10), KICH (Cor = 0.542, P = 3.15e-06) and LIHC (Cor = 0.669, P = 3.43e-46). As shown in **Figure 4E**, PHF19 expression was also significantly associated with the infiltration levels of CD4+ Th2 cells in ACC (Cor = 0.685, P = 2.27e-11), BLCA (Cor = 0.629, P = 6.29e-42), BRCA (Cor = 0.51, P = 7.19e-67), HNSC (Cor = 0.506, P = 2.70e-33), HNSC-HPV- (Cor = 0.506, P = 1.89e-27), KICH (Cor = 0.545, P = 2.72e-06), LIHC (Cor = 0.593, P = 3.37e-34), LUAD (Cor = 0.51, P = 6.23e-34), MESO (Cor = 0.553, P = 3.99e-08), PAAD (Cor = 0.505, P = 1.88e-12), SARC (Cor = 0.551, P = 8.36e-21), and THYM (Cor = 0.706, P = 1.31e-18). The profiles illustrated that PHF19, to a certain extent, was engaged in the immune infiltration-related pathways and served a critical role in the immuno-oncological interactions.

### Enrichment Analysis of PHF19-Related Partners

**Figure 5:**
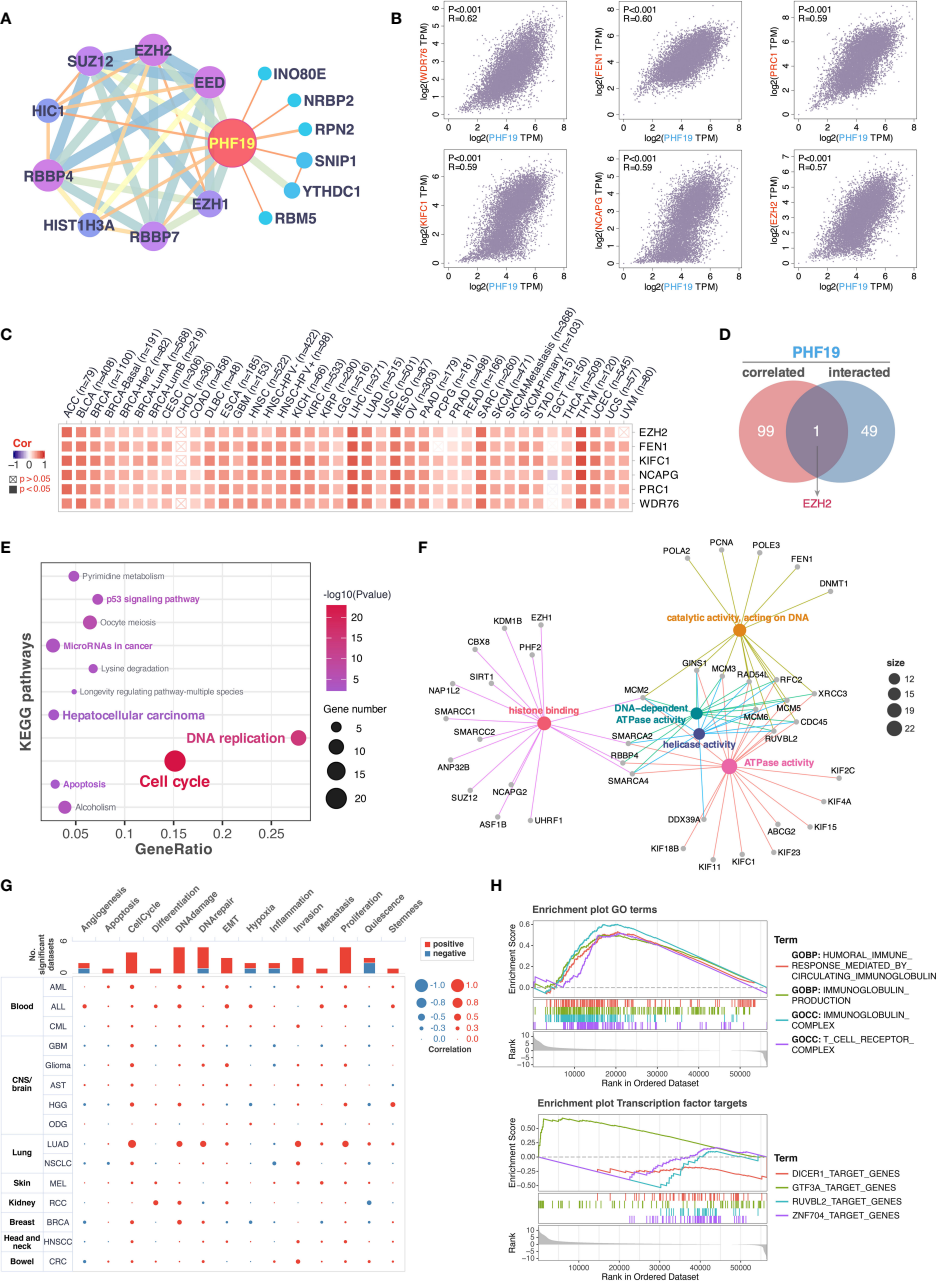
Enrichment analysis of PHF19-related partners. **(A)** PPI network for PHF19 was constructed in Cytoscape. **(B)** The expression correlation between PHF19 and selected targeting genes, including WDR76, FEN1, PRC1, KIFC1, NCAPG, and EZH2. **(C)** The heatmap showed that PHF19 was positively related to the selected genes in TCGA cancers. **(D)** Venn diagram of PHF19-interacted and correlated genes. KEGG pathway **(E)** and GO molecular function **(F)** enrichment analyses were performed. **(G)** CancerSEA was utilized for single-cell analysis to determine the functions of PHF19. **(H)** GSEA analysis of PHF19-related signaling pathways in TCGA LIHC dataset.

To further decipher the underlying molecular mechanisms by which PHF19 contributes to carcinogenesis, we next investigated the available experimentally confirmed PHF19-binding proteins and PHF19 expression-associated genes for pathway enrichment analyses. In total, 50 PHF19-interacted proteins were retrieved from the STRING database by experimental evidence, and the PPI network of proteins with confidence level > 0.200 was presented as **Figure 5A**. We next acquired the top 100 genes that associated with PHF19 expression based on TCGA and GTEx datasets by utilizing the GEPIA2 database. As seen in **Figure 5B**, the PHF19 expression was significantly positively correlated with the expression of WDR76 (WD repeat domain 76) (R = 0.62), FEN1 (flap structure-specific endonuclease 1) (R = 0.60), PRC1 (protein regulator of cytokinesis 1) (R = 0.59), KIFC1 (kinesin family member C1) (R = 0.59), NCAPG (non-SMC condensin I complex subunit G) (R = 0.59), and EZH2 (enhancer of zeste 2 polycomb repressive complex 2 subunit) (R = 0.57) genes (all P-values < 0.001). The correlation heatmap showed that PHF19 was positively related to the above genes in the majority of TCGA cancers (**Figure 5C**). Besides, we performed the Venn intersection analysis between the two datasets described above and identified a common member, namely EZH2 (**Figure 5D**). Further, these two datasets were combined to perform KEGG pathway and GO molecular function (MF) enrichment analyses. As presented in **Figure 5E**, several pathways including “p53 signaling pathway”, “microRNAs in cancer”, “hepatocellular carcinoma”, “apoptosis”, “DNA replication” and “cell cycle” were revealed as the most significantly enriched KEGG pathways, indicating that PHF19 was crucially involved in the development and progression of cancers, especially HCC. Meanwhile, the GO-MF enrichment analysis confirmed that five terms were highly enriched, such as histone binding, catalytic activity acting on DNA, DNA-dependent ATPase activity, helicase activity and ATPase activity (**Figure 5F**). We also performed single-cell analysis by using CancerSEA database, and determined that PHF19 clearly stimulated a multitude of carcinogenic processes, including promotion of the cell cycle, DNA damage, epithelial to mesenchymal transition (EMT), invasion, and proliferation in different cancer cell types (**Figure 5G**).

To gain insight into the potential effect of PHF19 on HCC progression, we then downloaded the LIHC RNA-seq data from TCGA portal and performed GSEA analysis based on PHF19 expression level to identify the relevant pathways and underlying mechanisms. Enrichment score (ES) was calculated to compare the enrichment of genes in a ranked list. Our results indicated that PHF19 was significantly enriched in humoral immune response mediated by circulating immunoglobulin, immunoglobulin production, and pathways related to immunoglobulin complex and T cell receptor complex (**Figure 5H**). Moreover, we conducted the transcription factor target analysis, and found that DICER1 (dicer 1, ribonuclease III), GTF3A (general transcription factor IIIA), RUVBL2 (RuvB like AAA ATPase 2), and ZNF704 (zinc finger protein 704) were the main transcription factors participating in the PHF19-regulated pathways in HCC.

### Construction and Evaluation of Prognostic Risk-Score Model

For assessing the application of PHF19-associated functional gene sets in HCC prognosis, we entered the variables in **Figure 5F** into a univariable Cox proportional hazard regression to analyze the training set, namely the TCGA LIHC cohort. This strategy led to an optimal eleven-gene prognostic model in HCC, and the formula was applied to calculate the risk score of each patient, as follows: RiskScore = 0.081 ∗ KIF2C + 0.026 ∗ SMARCC1 – 0.005 ∗ ASF1B + 0.032 ∗ RBBP4 + 0.031 ∗ MCM6 – 0.012 ∗ KIF11 – 0.089 ∗ RAD54L + 0.069 ∗ GINS1 + 0.123 ∗ CBX8 + 0.001 ∗ ANP32B – 0.084 ∗ SUZ12. To better validate the robustness of the model, GSE14520 cohort was used as the independent external validation dataset.

**Figure 6:**
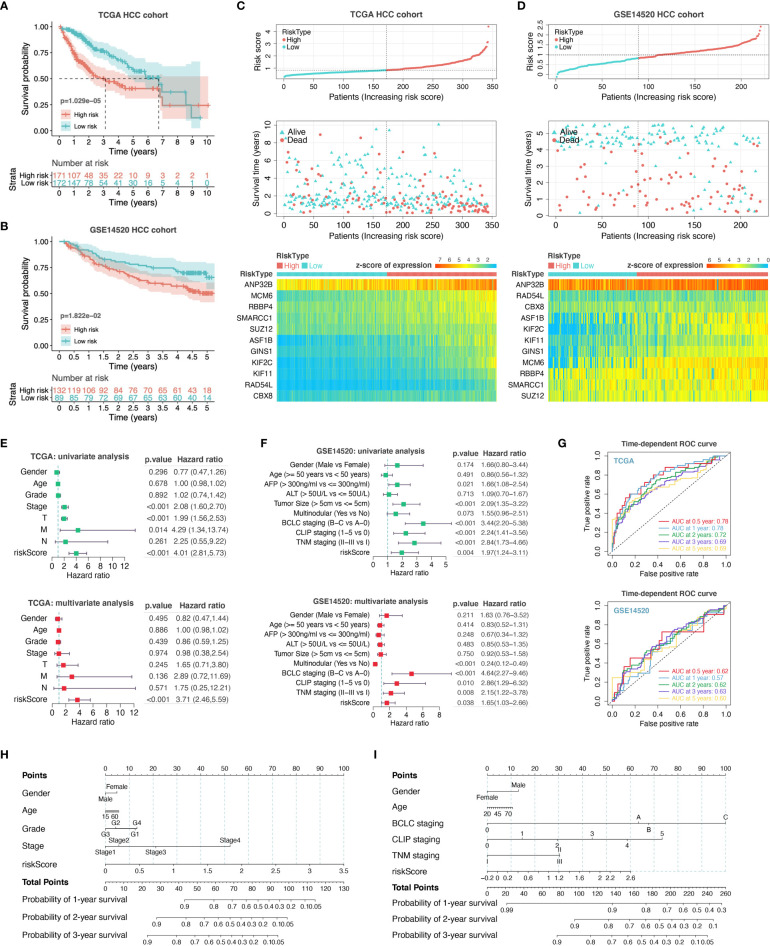
Construction and evaluation of prognostic risk-score model. We performed survival analyses between the high- and low-risk score groups in TCGA HCC cohort **(A)** and GSE14520 HCC cohort **(B)**. The distribution of risk scores, survival statuses, and signature gene expression levels for HCC patients in training **(C)** and validation sets **(D)** were visualized. Univariate and multivariate Cox regression analyses were conducted for each clinical feature and risk-score model in TCGA dataset **(E)** and GSE14520 dataset **(F)**. T, T stage; M, M stage; N, N stage; riskScore, risk-score model. **(G)** Time-dependent ROC curve analysis to assess the predictive efficacy of the prognostic signature. **(H, I)** Nomograms for quantitatively predicting the probability of 1-, 2-, and 3-year OS for HCC patients.

We first calculated and plotted the prognostic Kaplan-Meier survival curves predicted by this model in both internal and external datasets (**Figures 6A, B** **)**. The results showed that patients with high risk scores had obviously less survival probability than low-risk patients, which meant the higher the score, the worse the prognosis. The distribution of risk scores, survival statuses, and signature gene expression patterns for HCC patients in training and validation sets were visualized in **Figures 6C, D**, respectively. In TCGA LIHC cohort, both univariate (HR = 4.01, p < 0.001) and multivariate (HR = 3.71, p < 0.001) Cox regression analyses determined that the prognostic signature was strongly associated with prognosis (**Figure 6E**). Moreover, as shown in **Figure 6F**, the risk score was correlated with prognosis in univariate COX regression model (HR = 1.97, p = 0.004) in GSE14520 cohort, and the multivariate analysis suggested that the risk score was capable to independently predict the prognosis of HCC after adjusting for gender, age, AFP, ALT, tumor size, multinodular, BCLC staging, CLIP staging, and TNM staging (HR = 1.65, p = 0.038). These results suggested that the eleven-gene prognostic signature performed well in predicting the prognosis of HCC patients, and could function as a useful tool to supplement the gold standard for clinical diagnosis. Additionally, we further performed the time-dependent ROC curve analysis to validate the predictive classification efficiencies of risk-score model in HCC, and the area under the curve (AUC) values for 0.5-, 1-, 2-, 3-, and 5-years overall survival were presented in **Figure 6G**. Finally, as shown in **Figures 6H, I**, we formulated the prognostic nomograms to anticipate the individualized survival probability based on TCGA LIHC cohort and GSE14520 cohort, which might contribute to efficacy assessment and managing patients.

### Validation of PHF19 Expression and Impacts of PHF19 on Immune Infiltrates in HCC

**Figure 7:**
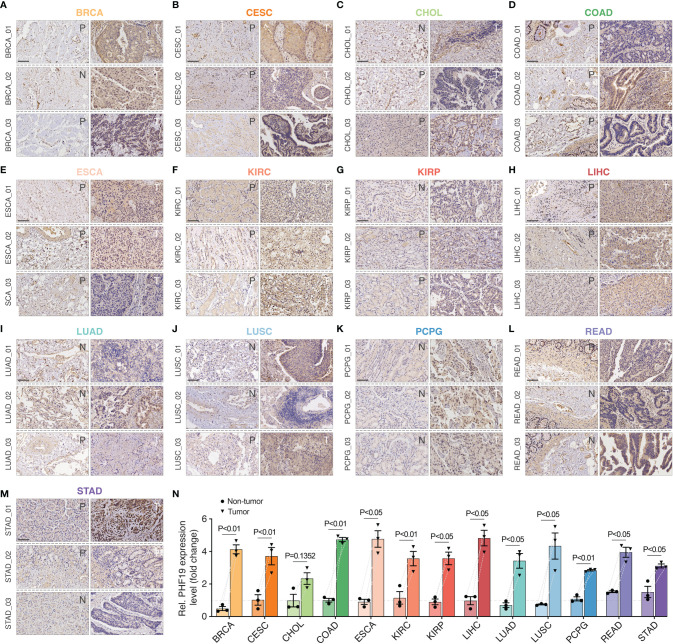
Immunohistochemical stainings of PHF19 in cancers. We detected the PHF19 protein expressions in BRCA, CESC, CHOL, COAD, ESCA, KIRC, KIRP, LIHC, LUAD, LUSC, PCPG, READ, and STAD **(A–M)** (N, distant noncancerous tissues from the surgical margin; P, adjacent noncancerous tissues from the surgical margin; T, tumor tissues). **(N)** The results were then quantitated. Data represent mean ± SEM.

To ensure positive confirmation of pathophysiological roles of PHF19, we applied experimental validation to investigate its clinicopathological characteristics. We performed immunohistochemical (IHC) analyses in 78 cancer samples across BRCA, CESC, CHOL, COAD, ESCA, KIRC, KIRP, LIHC, LUAD, LUSC, PCPG, READ, and STAD, with three pairs of different surgical specimens analyzed per tumor type (**Figures 7A** **–M**). Adjacent or distant noncancerous tissues from the surgical margin were used as the control tissues. We found that the PHF19 protein expressions were significantly higher in tumor tissues in comparison with the control tissues, and the results were quantitated in **Figure 7N**, which indicated the extensive carcinogenic effects of PHF19.

**Figure 8:**
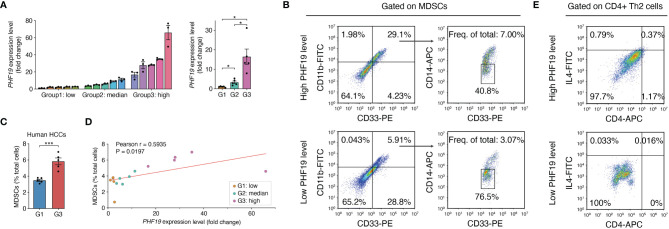
PHF19 correlates with the immune infiltrates of MDSCs and Th2 subsets of CD4+ T cells in HCC. **(A)** PHF19 mRNA expression levels of HCC tissues from 15 patients, and patients were divided into low- (G1), median- (G2) and high-PHF19 expression (G3) groups. **(B)** Representative flow cytometric analysis of MDSCs proportion in specimens. **(C)** The degree of MDSC immune infiltration was higher in G3 group. **(D)** MDSCs proportions are proportional to the PHF19 levels. **(E)** Representative flow cytometric analysis of CD4+ Th2 cells proportion in specimens.

In view of the prognostic value of PHF19 in HCC, we further studied the impacts of PHF19 expression on immune infiltration, by performing flow cytometry analysis on 15 clinical specimens diagnosed as HCC. MDSCs are a heterogeneous population of cells which expand during cancer, inflammation and infection, with a remarkable ability to suppress T-cell responses, and were defined as CD11b^+^ CD14^-^ CD33^+^ in most tumors (41). We first determined the PHF19 mRNA levels in all samples, and patients were ranked according to PHF19 expression, and divided into low-, median- and high-PHF19 expression groups, respectively (**Figure 8A**). Significant differences were observed between these three groups. We found that the MDSC infiltration ratios were clearly higher in high-PHF19 expression group than in low-PHF19 group (**Figures 8B, C** **)**, and PHF19 expression level was closely related to the degree of immune infiltration of MDSCs (**Figure 8D**). Similarly, we detected the infiltration of Th2 subsets of CD4+ T cells in specimens, which were defined as CD4^+^ IL4^+^. The results showed that hardly Th2 subsets can be detected in low-PHF19 expression group (**Figure 8E**). Collectively, these data indicated that PHF19 expression had noticeable effects on immune cell infiltration in HCC.

## Discussion

The physiological functions of PRC2 complex are subjected to intricate cellular regulation, which is correlated with the enormous complexity of PRC2 components (42). Previous studies have proved that H3K36me3-binding activity is harbored in the Tudor motif of PRC2-associated PCL protein named PHF19, and the Tudor function of PHF19 is also essential for H3K27me3 and repression of previously described ‘poised’ developmental genes (43). Although investigators have gained some understanding of the regulation of Polycomb activity by PHF19, little is known about whether and how it drives tumor initiation, progression, and metastasis (13). Collectively, as a critical epigenetic related gene, PHF19’s potential roles in carcinogenesis and cancer development are worthwhile to be disclosed.

In the present study, we explored the pan-cancer expression profiles of PHF19, and the correlation between PHF19 aberrant expression and patient prognosis in different cancers. Compared with corresponding noncancerous tissues, PHF19 expression was significantly up-regulated across a range of cancers, which implied the extensively oncogenic characteristics of PHF19 in cancers and promising perspectives in the field of cancer research. This present result is consistent with findings of previous study in 2004 when PHF19 was first identified in human tissues and extends the work in important ways (44). COX regression analyses suggested that elevated PHF19 expression may lead to shorter OS and DSS in ACC, KICH, KIRC, LGG, LIHC, MESO, and PCPG, and Kaplan-Meier analyses revealed that high PHF19 expression predicted worse OS in BC, KIRC, KIRP, LIHC, LUAD, LUSC, and UCEC. Using Mantel-Cox test through the GEPIA2 database, we further validated that PHF19 overexpression was related to unfavorable DFS of ACC, LGG, LIHC, and UVM. Notably, these results particularly revealed PHF19 as a carcinogenic indicator of HCC prognosis, regardless of the prognostic algorithm. We further performed the enrichment analyses and identified that PHF19 was significantly enriched in cell cycle pathways, and related to the progression of HCC. Consistent with our results, past studies have shown that PHF19 knockdown resulted in the reduction of growth and cell cycle arrest in multiple myeloma (18), and reduced PHF19 levels in chronic myeloid leukemia cells arrested the cell cycle and promoted differentiation toward erythroid fate (45).

Cancer is a complicated disease involving complex reciprocal networks between tumor cells and the immune system. TME is composed of a variety of cell types, including mesenchymal cells and resident and infiltrating immune cells (46). Our initial exploration demonstrated that aberrant PHF19 expression was correlated with increased immune cell infiltration of MDSCs and Th2 subset of CD4+ T cells in the majority of cancers, which implied potential value of clinical application for PHF19 in cancer treatment. Ample evidence has supported that MDSCs are critical in regulating immune responses under pathological conditions, and play an prominent role in tumor angiogenesis, drug resistance, and promotion of cancer metastases (47). Past literature pointed that the discovery of CD4+ T cell subset-defining key transcription factors and framing of the Th1/Th2 paradigm ignited the CD4+ T cell field (48). CD4+ T cell subsets, such as Th1, Th2, Th17, and regulatory T (Treg) cells, serve pivotal functions in cancer immunity, among which the Th2 subset of CD4+ T cells secretes IL-4, IL-5, and IL-13, and activates B cells to become antibody-secreting plasma cells (49). It is worth noting that the balance between Th1 and Th2 differentiation is critical for immune homeostasis, and shift of Th1/Th2 balance towards Th2 cells is correlated with the immunosuppression and progression of cancer (50–52). Previously, researchers found that PHF19 restrained T cell senescence and sustained CD8+ T cell antitumor responses by orchestrating a transcriptional program extensively shared with miR-155 (53). Yet, more exact mechanisms underpinning the effects of PHF19 on tumor immunity remain to be elucidated. Cancer immunotherapies, especially immune checkpoint blockade therapy, have shifted the treatment of cancer by promoting complete and durable responses, and are now standard treatment for various malignant tumors (54). Unfortunately, only a small proportion of patients with certain cancer types respond to immunotherapy, probably due to inadequate immune activation to recognize tumor-specific antigens (55). Therefore, it is essential to identify additional potential therapeutic targets. Our current research showed that PHF19 levels demonstrated strong correlations with a variety of immune checkpoint molecules in BLCA, BRCA, HNSC, LIHC, PRAD and THCA. Moreover, in LIHC cohort, PHF19 was closely correlated with checkpoints including BTLA, CD27, CD274, CD276, CD28, CD40, CD70, CD80, CD86, CTLA4, HAVCR2, HHLA2, ICOS, IDO1, LAG3, PDCD1, TIGIT, TNFRSF9, and TNFSF9, indicating that PHF19 serves as a potential immune-related therapeutic target for HCC patients. In consequence, the present study points new directions for delineating the relationships between the epigenetic related PHF19 gene and immune cell infiltration within the TME, which may have important implications for exploring new strategies for cancer therapy.

As the fourth leading cause of cancer-related mortality globally, HCC imposes a huge health burden on society (56). To better explore new targets for early diagnosis and treatment, there is an urgent need to determine novel prognostic predictors and construct more reliable prognostic models of HCC. Our results provided evidence that elevated PHF19 expression indicated worse clinical outcomes in HCC patients. The GSEA results revealed that PHF19 was associated with the cellular components including immunoglobulin complex and T cell receptor complex in HCC, which provided new ideas for future research. Moreover, after the generation of eleven-gene prognostic signature, we performed a preliminary *in silico* validation using the external GEO dataset, which proved the effectiveness of the model. Taken together, the present study unveiled the complicated roles of PHF19 aberrant expression in the progression and prognoses of cancers, and summarized the pivotal signaling pathways associated with the pathophysiological functions of this epigenetic related gene. We also demonstrated that PHF19 played important roles in regulating tumor-infiltration of immune cells, and might exhibit beneficial therapeutic effects on cancer treatment. Enrichment Analysis highlighted the potential mechanistic basis of PHF19 in induction of HCC development, and the prognostic signatures derived from PHF19-related functional gene sets were validated to predict the overall survival of HCC independently. While these findings warrant further investigation, our research provides novel insights into the promising application prospects of PHF19 in the field of cancer research.

## Data Availability Statement

Publicly available datasets were analyzed in this study. All relevant data can be found here: UCSC Xena (http://xena.ucsc.edu/), TCGA GDC Portal (https://portal.gdc.cancer.gov/), GSE14520 (https://www.ncbi.nlm.nih.gov/geo/query/acc.cgi?acc=GSE14520), Human Protein Atlas (https://www.proteinatlas.org), ONCOMINE (www.oncomine.org), TIMER2.0 (http://timer.cistrome.org/), GEPIA (http://gepia.cancer-pku.cn/index.html), GEPIA2 (http://gepia2.cancer-pku.cn), Kaplan-Meier plotter (https://kmplot.com/analysis/), cBioPortal (http://www.cbioportal.org/), STRING (https://www.string-db.org/), and CancerSEA (http://biocc.hrbmu.edu.cn/CancerSEA/).

## Ethics Statement

The studies involving human participants were reviewed and approved by the human ethics committees of the Affiliated Drum Tower Hospital, Medical School of Nanjing University. The patients/participants provided their written informed consent to participate in this study.

## Author Contributions

Z-yZ and N-T conceived and designed the study, collected and assembled data, performed data analysis and interpretation, and wrote the manuscript. M-fW and J-cZ collected and assembled data, and performed data analysis and interpretation. J-lW, H-zR and X-lS conceived and designed the study, provided financial support and study material, performed data analysis and interpretation, wrote and gave final approval of the manuscript. All authors read and approved the manuscript.

## Funding

This work was funded by the National Natural Science Foundation of China (81872359, to X-lS), Jiangsu Provincial Key Research and Development (BE2020752, to X-lS), Key Scientific Research Project of Jiangsu Provincial Health Commission (ZDA2020002, to X-lS), Jiangsu Province Natural Science Foundation (BK20190114, to J-lW), Jiangsu Province Postdoctoral Research Funding Program(2021K116B, to J-lW), the Nanjing Medical Science and Technique Development Foundation (QRX17129, to H-zR), the Nanjing health science and technology development project for Distinguished Young Scholars (JQX19002, to H-zR), the Fundamental Research Funds for the Central Universities (0214-14380510, to H-zR), the Nanjing health science and technology development project for Medical and health research (YKK19070, to J-lW), the Nanjing Science and technology project (201911039, to J-lW), Project of Modern Hospital Management and Development Institute, Nanjing University and Aid project of Nanjing Drum Tower Hospital Health, Education & Research Foundation(NDYG2020047, to J-lW), the Chen Xiao-ping Foundation for the Development of Science and Technology of Hubei Province, China (CXPJJH121001-2021073, to H-zR), the Innovation Capability Development Project of Jiangsu Province (No. BM2015004).

## Conflict of Interest

The authors declare that the research was conducted in the absence of any commercial or financial relationships that could be construed as a potential conflict of interest.

## Publisher’s Note

All claims expressed in this article are solely those of the authors and do not necessarily represent those of their affiliated organizations, or those of the publisher, the editors and the reviewers. Any product that may be evaluated in this article, or claim that may be made by its manufacturer, is not guaranteed or endorsed by the publisher.

## References

[1] Xu Z Zeng S Gong Z Yan Y. Exosome-Based Immunotherapy: A Promising Approach for Cancer Treatment. Mol Cancer (2020) 19(1):160. doi: 10.1186/s12943-020-01278-3 33183286 PMC7661275

[2] Manzella G Schreck LD Breunis WB Molenaar J Merks H Barr FG. Phenotypic Profiling With a Living Biobank of Primary Rhabdomyosarcoma Unravels Disease Heterogeneity and AKT Sensitivity. Nat Commun (2020) 11(1):4629. doi: 10.1038/s41467-020-18388-7 32934208 PMC7492191

[3] Yang Z Wu L Wang A Tang W Zhao Y Zhao H. dbDEMC 2.0: Updated Database of Differentially Expressed miRNAs in Human Cancers. Nucleic Acids Res (2017) 45(D1):D812–d8. doi: 10.1093/nar/gkw1079 PMC5210560 27899556

[4] Cieslik M Chinnaiyan AM. Global Genomics Project Unravels Cancer’s Complexity at Unprecedented Scale. Nature (2020) 578(7793):39–40. doi: 10.1038/d41586-020-00213-2 32025004

[5] Abed JA Jones RS. H3K36me3 Key to Polycomb-Mediated Gene Silencing in Lineage Specification. Nat Struct Mol Biol (2012) 19(12):1214–5. doi: 10.1038/nsmb.2458 PMC4019213 23211767

[6] Rose NR King HW Blackledge NP Fursova NA Ember KJ Fischer R. RYBP Stimulates PRC1 to Shape Chromatin-Based Communication Between Polycomb Repressive Complexes. Elife (2016) 5:e18591. doi: 10.7554/eLife.18591 27705745 PMC5065315

[7] Wang W Qin JJ Voruganti S Nag S Zhou J Zhang R. Polycomb Group (PcG) Proteins and Human Cancers: Multifaceted Functions and Therapeutic Implications. Med Res Rev (2015) 35(6):1220–67. doi: 10.1002/med.21358 PMC4718713 26227500

[8] Di Croce L Helin K. Transcriptional Regulation by Polycomb Group Proteins. Nat Struct Mol Biol (2013) 20(10):1147–55. doi: 10.1038/nsmb.2669 24096405

[9] Sanna L Marchesi I Melone MAB Bagella L. The Role of Enhancer of Zeste Homolog 2: From Viral Epigenetics to the Carcinogenesis of Hepatocellular Carcinoma. J Cell Physiol (2018) 233(9):6508–17. doi: 10.1002/jcp.26545 PMC13482477 29574790

[10] Laugesen A Højfeldt JW Helin K. Molecular Mechanisms Directing PRC2 Recruitment and H3K27 Methylation. Mol Cell (2019) 74(1):8–18. doi: 10.1016/j.molcel.2019.03.011 30951652 PMC6452890

[11] Duncan IM. Polycomblike: A Gene That Appears to be Required for the Normal Expression of the Bithorax and Antennapedia Gene Complexes of Drosophila Melanogaster. Genetics (1982) 102(1):49–70. doi: 10.1093/genetics/102.1.49 6813190 PMC1201924

[12] Hunkapiller J Shen Y Diaz A Cagney G McCleary D Ramalho-Santos M. Polycomb-Like 3 Promotes Polycomb Repressive Complex 2 Binding to CpG Islands and Embryonic Stem Cell Self-Renewal. PloS Genet (2012) 8(3):e1002576. doi: 10.1371/journal.pgen.1002576 22438827 PMC3305387

[13] Li H Liefke R Jiang J Kurland JV Tian W Deng P. Polycomb-Like Proteins Link the PRC2 Complex to CpG Islands. Nature (2017) 549(7671):287–91. doi: 10.1038/nature23881 PMC5937281 28869966

[14] Ruan S Zhang H Tian X Zhang Z Huang H Shi C. PHD Finger Protein 19 Enhances the Resistance of Ovarian Cancer Cells to Compound Fuling Granule by Protecting Cell Growth, Invasion, Migration, and Stemness. Front Pharmacol (2020) 11:150. doi: 10.3389/fphar.2020.00150 32180719 PMC7059104

[15] Dong C Nakagawa R Oyama K Yamamoto Y Zhang W Dong A. Structural Basis for Histone Variant H3tK27me3 Recognition by PHF1 and PHF19. Elife (2020) 9:e58675. doi: 10.7554/eLife.58675 32869745 PMC7492083

[16] Brien GL Gambero G O’Connell DJ Jerman E Turner SA Egan CM. Polycomb PHF19 Binds H3K36me3 and Recruits PRC2 and Demethylase NO66 to Embryonic Stem Cell Genes During Differentiation. Nat Struct Mol Biol (2012) 19(12):1273–81. doi: 10.1038/nsmb.2449 23160351

[17] Ballaré C Lange M Lapinaite A Martin GM Morey L Pascual G. Phf19 Links Methylated Lys36 of Histone H3 to Regulation of Polycomb Activity. Nat Struct Mol Biol (2012) 19(12):1257–65. doi: 10.1038/nsmb.2434 PMC3926938 23104054

[18] Mason MJ Schinke C Eng CLP Towfic F Gruber F Dervan A. Multiple Myeloma DREAM Challenge Reveals Epigenetic Regulator PHF19 as Marker of Aggressive Disease. Leukemia (2020) 34(7):1866–74. doi: 10.1038/s41375-020-0742-z PMC7326699 32060406

[19] Wang H Xu P Sun G Lv J Cao J Xu Z. Downregulation of PHF19 Inhibits Cell Growth and Migration in Gastric Cancer. Scand J Gastroenterol (2020) 55(6):687–93. doi: 10.1080/00365521.2020.1766555 32449434

[20] Deng Q Hou J Feng L Lv A Ke X Liang H. PHF19 Promotes the Proliferation, Migration, and Chemosensitivity of Glioblastoma to Doxorubicin Through Modulation of the SIAH1/β-Catenin Axis. Cell Death Dis (2018) 9(11):1049. doi: 10.1038/s41419-018-1082-z 30323224 PMC6189144

[21] Li P Sun J Ruan Y Song L. High PHD Finger Protein 19 (PHF19) Expression Predicts Poor Prognosis in Colorectal Cancer: A Retrospective Study. PeerJ (2021) 9:e11551. doi: 10.7717/peerj.11551 34141488 PMC8176917

[22] Hutter C Zenklusen JC. The Cancer Genome Atlas: Creating Lasting Value Beyond Its Data. Cell (2018) 173(2):283–5. doi: 10.1016/j.cell.2018.03.042 29625045

[23] Goldman MJ Craft B Hastie M Repečka K McDade F Kamath A. Visualizing and Interpreting Cancer Genomics Data *via* the Xena platform. Nat Biotechnol (2020) 38(6):675–8. doi: 10.1038/s41587-020-0546-8 PMC7386072 32444850

[24] Roessler S Jia HL Budhu A Forgues M Ye QH Lee JS. A Unique Metastasis Gene Signature Enables Prediction of Tumor Relapse in Early-Stage Hepatocellular Carcinoma Patients. Cancer Res (2010) 70(24):10202–12. doi: 10.1158/0008-5472.Can-10-2607 PMC3064515 21159642

[25] Uhlén M Fagerberg L Hallström BM Lindskog C Oksvold P Mardinoglu A. Proteomics. Tissue-Based Map of the Human Proteome. Science (2015) 347(6220):1260419. doi: 10.1126/science.1260419 25613900

[26] Rhodes DR Yu J Shanker K Deshpande N Varambally R Ghosh D. ONCOMINE: A Cancer Microarray Database and Integrated Data-Mining Platform. Neoplasia (2004) 6(1):1–6. doi: 10.1016/s1476-5586(04)80047-2 15068665 PMC1635162

[27] Li T Fu J Zeng Z Cohen D Li J Chen Q. TIMER2.0 for Analysis of Tumor-Infiltrating Immune Cells. Nucleic Acids Res (2020) 48(W1):W509–14. doi: 10.1093/nar/gkaa407 PMC7319575 32442275

[28] Tang Z Li C Kang B Gao G Li C Zhang Z. GEPIA: A Web Server for Cancer and Normal Gene Expression Profiling and Interactive Analyses. Nucleic Acids Res (2017) 45(W1):W98–w102. doi: 10.1093/nar/gkx247 28407145 PMC5570223

[29] Nagy Á Munkácsy G Győrffy B. Pancancer Survival Analysis of Cancer Hallmark Genes. Sci Rep (2021) 11(1):6047. doi: 10.1038/s41598-021-84787-5 33723286 PMC7961001

[30] Tang Z Kang B Li C Chen T Zhang Z. GEPIA2: An Enhanced Web Server for Large-Scale Expression Profiling and Interactive Analysis. Nucleic Acids Res (2019) 47(W1):W556–60. doi: 10.1093/nar/gkz430 PMC6602440 31114875

[31] Gao J Aksoy BA Dogrusoz U Dresdner G Gross B Sumer SO. Integrative Analysis of Complex Cancer Genomics and Clinical Profiles Using the Cbioportal. Sci Signal (2013) 6(269):pl1. doi: 10.1126/scisignal.2004088 23550210 PMC4160307

[32] Yoshihara K Shahmoradgoli M Martínez E Vegesna R Kim H Torres-Garcia W. Inferring Tumour Purity and Stromal and Immune Cell Admixture From Expression Data. Nat Commun (2013) 4:2612. doi: 10.1038/ncomms3612 24113773 PMC3826632

[33] Zhao Y Zhang M Pu H Guo S Zhang S Wang Y. Prognostic Implications of Pan-Cancer CMTM6 Expression and Its Relationship With the Immune Microenvironment. Front Oncol (2020) 10:585961. doi: 10.3389/fonc.2020.585961 33552963 PMC7855963

[34] Szklarczyk D Gable AL Lyon D Junge A Wyder S Huerta-Cepas J. STRING V11: Protein-Protein Association Networks With Increased Coverage, Supporting Functional Discovery in Genome-Wide Experimental Datasets. Nucleic Acids Res (2019) 47(D1):D607–13. doi: 10.1093/nar/gky1131 PMC6323986 30476243

[35] Otasek D Morris JH Bouças J Pico AR Demchak B. Cytoscape Automation: Empowering Workflow-Based Network Analysis. Genome Biol (2019) 20(1):185. doi: 10.1186/s13059-019-1758-4 31477170 PMC6717989

[36] Zhou Y Zhou B Pache L Chang M Khodabakhshi AH Tanaseichuk O. Metascape Provides a Biologist-Oriented Resource for the Analysis of Systems-Level Datasets. Nat Commun (2019) 10(1):1523. doi: 10.1038/s41467-019-09234-6 30944313 PMC6447622

[37] Yuan H Yan M Zhang G Liu W Deng C Liao G. CancerSEA: A Cancer Single-Cell State Atlas. Nucleic Acids Res (2019) 47(D1):D900–8. doi: 10.1093/nar/gky939 PMC6324047 30329142

[38] Chang Y Kang SY Kim J Kang HR Kim HY. Functional Defects in Type 3 Innate Lymphoid Cells and Classical Monocytes in a Patient With Hyper-IgE Syndrome. Immune Netw (2017) 17(5):352–64. doi: 10.4110/in.2017.17.5.352 PMC5662784 29093656

[39] Shu L Liu Y Li J Wu X Li Y Huang H. Landscape Profiling Analysis of DPP4 in Malignancies: Therapeutic Implication for Tumor Patients With Coronavirus Disease 2019. Front Oncol (2021) 11:624899. doi: 10.3389/fonc.2021.624899 33614513 PMC7890191

[40] Mo X Huang X Feng Y Wei C Liu H Ru H. Immune Infiltration and Immune Gene Signature Predict the Response to Fluoropyrimidine-Based Chemotherapy in Colorectal Cancer Patients. Oncoimmunology (2020) 9(1):1832347. doi: 10.1080/2162402x.2020.1832347 33117604 PMC7575007

[41] Gabrilovich DI Nagaraj S. Myeloid-Derived Suppressor Cells as Regulators of the Immune System. Nat Rev Immunol (2009) 9(3):162–74. doi: 10.1038/nri2506 PMC2828349 19197294

[42] Schuettengruber B Bourbon HM Di Croce L Cavalli G. Genome Regulation by Polycomb and Trithorax: 70 Years and Counting. Cell (2017) 171(1):34–57. doi: 10.1016/j.cell.2017.08.002 28938122

[43] Cai L Rothbart SB Lu R Xu B Chen WY Tripathy A. An H3K36 Methylation-Engaging Tudor Motif of Polycomb-Like Proteins Mediates PRC2 Complex Targeting. Mol Cell (2013) 49(3):571–82. doi: 10.1016/j.molcel.2012.11.026 PMC3570589 23273982

[44] Wang S Robertson GP Zhu J. A Novel Human Homologue of Drosophila Polycomblike Gene Is Up-Regulated in Multiple Cancers. Gene (2004) 343(1):69–78. doi: 10.1016/j.gene.2004.09.006 15563832

[45] García-Montolio M Ballaré C Blanco E Gutiérrez A Aranda S Gómez A. Polycomb Factor PHF19 Controls Cell Growth and Differentiation Toward Erythroid Pathway in Chronic Myeloid Leukemia Cells. Front Cell Dev Biol (2021) 9:655201. doi: 10.3389/fcell.2021.655201 33996816 PMC8116664

[46] Cioni B Zaalberg A van Beijnum JR Melis MHM van Burgsteden J Muraro MJ. Androgen Receptor Signalling in Macrophages Promotes TREM-1-Mediated Prostate Cancer Cell Line Migration and Invasion. Nat Commun (2020) 11(1):4498. doi: 10.1038/s41467-020-18313-y 32908142 PMC7481219

[47] Gabrilovich DI. Myeloid-Derived Suppressor Cells. Cancer Immunol Res (2017) 5(1):3–8. doi: 10.1158/2326-6066.Cir-16-0297 28052991 PMC5426480

[48] Ruterbusch M Pruner KB Shehata L Pepper M. *In Vivo* CD4(+) T Cell Differentiation and Function: Revisiting the Th1/Th2 Paradigm. Annu Rev Immunol (2020) 38:705–25. doi: 10.1146/annurev-immunol-103019-085803 32340571

[49] Li Q Zou J Wang M Ding X Chepelev I Zhou X. Critical Role of Histone Demethylase Jmjd3 in the Regulation of CD4+ T-Cell Differentiation. Nat Commun (2014) 5:5780. doi: 10.1038/ncomms6780 25531312 PMC4274750

[50] Ao L Shi J Bai Y Zhang S Gan J. Effects of Transcutaneous Electrical Acupoint Stimulation on Perioperative Immune Function and Postoperative Analgesia in Patients Undergoing Radical Mastectomy: A Randomized Controlled Trial. Exp Ther Med (2021) 21(3):184. doi: 10.3892/etm.2021.9615 33488793 PMC7812592

[51] Wang Z Sokolovska A Seymour R Sundberg JP Hogenesch H. SHARPIN Is Essential for Cytokine Production, NF-κb Signaling, and Induction of Th1 Differentiation by Dendritic Cells. PloS One (2012) 7(2):e31809. doi: 10.1371/journal.pone.0031809 22348129 PMC3279418

[52] Kantola T Klintrup K Väyrynen JP Vornanen J Bloigu R Karhu T. Stage-Dependent Alterations of the Serum Cytokine Pattern in Colorectal Carcinoma. Br J Cancer (2012) 107(10):1729–36. doi: 10.1038/bjc.2012.456 PMC3493870 23059742

[53] Ji Y Fioravanti J Zhu W Wang H Wu T Hu J. miR-155 Harnesses Phf19 to Potentiate Cancer Immunotherapy Through Epigenetic Reprogramming of CD8(+) T Cell Fate. Nat Commun (2019) 10(1):2157. doi: 10.1038/s41467-019-09882-8 31089138 PMC6517388

[54] Song W Shen L Wang Y Liu Q Goodwin TJ Li J. Synergistic and Low Adverse Effect Cancer Immunotherapy by Immunogenic Chemotherapy and Locally Expressed PD-L1 Trap. Nat Commun (2018) 9(1):2237. doi: 10.1038/s41467-018-04605-x 29884866 PMC5993831

[55] Lv M Chen M Zhang R Zhang W Wang C Zhang Y. Manganese Is Critical for Antitumor Immune Responses *via* cGAS-STING and Improves the Efficacy of Clinical Immunotherapy. Cell Res (2020) 30(11):966–79. doi: 10.1038/s41422-020-00395-4 PMC7785004 32839553

[56] Behary J Amorim N Jiang XT Raposo A Gong L McGovern E. Gut Microbiota Impact on the Peripheral Immune Response in Non-Alcoholic Fatty Liver Disease Related Hepatocellular Carcinoma. Nat Commun (2021) 12(1):187. doi: 10.1038/s41467-020-20422-7 33420074 PMC7794332

